# Dried Distillers’ Grains with Solubles in Ruminant Diets: Effects on Enteric Methane Emissions and Production Responses—A Systematic Review

**DOI:** 10.3390/ani16152427

**Published:** 2026-08-05

**Authors:** Lwazi Mwanda, Lwando Mbambalala, Musa Ikramatu, Lindokuhle C. Mhlongo, Róbert Tóthi

**Affiliations:** 1 Department of Animal and Wildlife Sciences, University of Pretoria, Pretoria 0028, South Africa; 2 Agricultural Research Council Animal Production, Irene, P/Bag X02, Centurion 0062, South Africa; 3 School of Interdisciplinary Research and Graduate Studies, College of Graduate Studies, University of South Africa, Preller Street, Muckleneuk Ridge, Pretoria 0003, South Africa; dr.mbambalala@gmail.com; 4 Institute of Physiology and Nutrition, Department of Farm Animal Nutrition, Hungarian University of Agriculture and Life Sciences Kaposvár Campus, Guba Sándor Str. 40., H-7400 Kaposvár, Hungary; ikramahkam@gmail.com (M.I.); tothi.robert@uni-mate.hu (R.T.); 5 Department of Animal Science, University of the Free State, Bloemfontein 9300, South Africa; mhlongolc1@ufs.ac.za

**Keywords:** feed efficiency, rumen fermentation, digestibility, metabolizable protein, nitrogen utilization, methane intensity, dietary lipids, livestock sustainability

## Abstract

Enteric methane (CH_4_) emissions from ruminants contribute to agricultural greenhouse gas emissions and represent a loss of dietary energy that could otherwise support animal production. Dried distillers’ grains with solubles (DDGSs) have received increasing attention as an alternative feed ingredient because of their nutritional value and potential to influence rumen fermentation, nutrient utilization, and CH_4_ emissions. This systematic review evaluated the effects of DDGSs on enteric CH_4_ emissions and nutrient utilization in ruminant diets. Literature searches were conducted in Google Scholar, Scopus, and Web of Science for studies published between 2010 and 2025. Following PRISMA guidelines and using the SYRCLE risk of bias tool, 17 studies, comprising 9 in vivo, 5 in vitro, and 3 integrated in vivo–in vitro studies, were included in the qualitative synthesis. The DDGS supplementation did not consistently reduce absolute CH_4_ production, although reductions in CH_4_ yield and emission intensity were more frequently reported. Moderate inclusion levels (up to 20% of diet dry matter) were frequently associated with improved nutrient utilization, feed efficiency, and animal performance, although these responses varied among studies. Reductions in CH_4_ yield and emission intensity were more commonly reported than reductions in absolute CH_4_ production. Higher inclusion levels (>30% of diet dry matter) produced more variable responses and were often associated with reduced fiber digestibility. In conclusion, the available evidence suggests that DDGS may act as a dietary modifier capable of improving nutrient utilization and production efficiency under appropriate dietary conditions; however, responses remain dependent on inclusion level, DDGS source, basal diet composition, and production system.

## Simple Summary

Ruminants release methane (CH_4_) during digestion, which contributes to climate change and represents a loss of feed energy that could otherwise support animal production. Dried distillers’ grains with solubles (DDGSs), a feed ingredient produced during ethanol manufacturing, are increasingly used in livestock diets because they provide protein, energy, and other nutrients. This review examined published research to determine whether including this ingredient in ruminant diets can reduce CH_4_ emissions while maintaining animal performance. Seventeen studies (9 in vivo, 5 in vitro, and 3 integrated in vivo–in vitro studies) were evaluated. The findings showed that DDGS did not consistently reduce total CH_4_ production. However, they often improved the efficiency with which animals converted feed into milk or meat, resulting in lower CH_4_ emissions per unit of product. Moderate DDGS inclusion levels were frequently associated with improved feed efficiency and animal performance, although these responses were not consistent across all studies. Higher inclusion levels generally produced more variable responses and, in some cases, reduced fiber digestibility. The effects varied depending on the amount included in the diet, the source of the ingredient, and the type of feed being replaced. These findings suggest that DDGSs can support more efficient livestock production and may help reduce the environmental impact of ruminant farming when used appropriately.

## 1. Introduction

Atmospheric methane (CH_4_) remains a major contributor to anthropogenic greenhouse gas (GHG) emissions, with enteric fermentation from ruminants representing one of the largest biological sources [1,2]. As livestock production intensifies globally, increasing attention has focused on nutritional strategies capable of improving feed efficiency while reducing CH_4_ emissions. Among these, dried distillers’ grains with solubles (DDGSs), a co-product of ethanol production, have emerged as an important alternative feed resource in ruminant nutrition because of their high concentrations of crude protein, digestible fiber, fat, and phosphorus [3,4]. The growing use of DDGSs in ruminant diets reflects both their nutritional value and economic importance within grain-processing industries. Despite growing interest in DDGS as a dietary intervention, CH_4_ responses remain inconsistent across studies. For instance, some studies report reductions in CH_4_ yield or CH_4_ intensity following DDGS supplementation [5,6,7], whereas others show limited effects or increases in absolute CH_4_ production [8,9]. This inconsistency reflects the highly variable chemical composition of DDGS, which differs according to grain source, fermentation efficiency, oil extraction, and processing conditions. In particular, DDGS composition can vary significantly in lipid, neutral detergent fiber (NDF), and rumen undegradable protein concentrations, all of which influence rumen fermentation pathways and hydrogen (H_2_) balance [10,11].

Mechanistically, the CH_4_ response to DDGS is closely linked to how these compositional fractions alter ruminal fermentation. The lipid fraction of corn DDGS is dominated by unsaturated fatty acids, particularly linoleic acid (C18:2 n-6), which represented 53.7% of total fatty acids, whereas oleic acid (C18:1) accounted for 25.6% in DDGSs obtained from three corn ethanol plants [12]. During ruminal biohydrogenation, linoleic acid undergoes sequential hydrogenation to more saturated fatty acids, incorporating reducing equivalents into the hydrogenation process [13,14]. Because methanogens also require H_2_ to reduce CO_2_ to CH_4_, biohydrogenation may decrease the availability of metabolic hydrogen for methanogenesis [15]. Consequently, the high linoleic acid content of corn DDGS may contribute to reduced CH_4_ production by competing with methanogens for reducing equivalents during fatty acid hydrogenation. In addition, linoleic acid and other polyunsaturated fatty acids (PUFAs) can modify the ruminal microbial community by serving as substrates for biohydrogenating bacteria while inhibiting the growth of susceptible ruminal microorganisms, particularly cellulolytic bacteria and fungi [16]. The antimicrobial activity of unsaturated fatty acids has also been attributed to their ability to disrupt microbial cell membrane structure, increasing membrane permeability and impairing cellular function, with Gram-positive cellulolytic bacteria being particularly susceptible [16,17]. Furthermore, dietary PUFAs may suppress ciliated ruminal protozoa, thereby indirectly reducing methanogenesis because many methanogenic archaea occur as endo- or ectosymbionts of protozoa and utilize protozoa-derived H_2_ through interspecies hydrogen transfer [18,19]. In contrast, the fiber fraction becomes concentrated during ethanol production and promotes acetate formation and metabolic H_2_ release during ruminal fermentation, thereby providing substrate for methanogenesis [11]. Consequently, the net effect of corn DDGS on CH_4_ production depends on the balance between lipid-mediated inhibition of methanogenesis and H_2_ generation associated with fermentation of the concentrated fiber fraction [11,18]. These contrasting mechanisms also influence rumen fermentation and nutrient utilization. Moderate inclusion levels (up to approximately 20% of dietary DM) generally improve metabolizable protein supply, feed efficiency, and animal performance, particularly in protein- or energy-deficient production systems [20,21]. However, higher inclusion levels (30–40% of dietary DM) may impair fiber digestion and alter rumen fermentation because of increased dietary lipid concentrations and reduced effective fiber supply [22,23].

Although research evaluating DDGS in ruminant diets has increased considerably, current evidence remains fragmented across studies differing in animal species, basal diet composition, DDGS source, and CH_4_ reporting metrics. In addition, CH_4_ responses are frequently interpreted independently of associated changes in rumen fermentation and nutrient utilization, limiting mechanistic understanding of the factors driving variability in outcomes. Recent studies have expanded beyond conventional evaluations of rumen fermentation by incorporating high-precision CH_4_ phenotyping techniques, including GreenFeed systems [9], indirect and respiration calorimetry [7,19], and rumen microbial community profiling using next-generation sequencing [24,25]. These methodological advances have generated new evidence linking dietary composition with rumen microbial ecology, nutrient utilization, and enteric CH_4_ emissions. However, this recent evidence has not been systematically synthesized to explain why DDGS frequently reduces CH_4_ yield or emission intensity while producing inconsistent responses in absolute CH_4_ production. Although a recent systematic review and meta-analysis by Malik et al. [26] evaluated the effects of DDGS supplementation on enteric CH_4_ emissions in dairy and beef cattle, its scope was limited to in vivo studies and focused primarily on CH_4_ production, CH_4_ yield, and dry matter intake. The present review expands the current evidence by synthesizing findings from in vivo, in vitro, and integrated studies across ruminant species. Specifically, this comprehensive approach enables an evaluation of the effects of DDGS on CH_4_ emissions and production responses while also examining the influence of DDGS inclusion level, source, basal diet composition, and nutrient substitution patterns on the variability in reported responses.

## 2. Materials and Methods

### 2.1. Review Protocol and Reporting Guidelines (PRISMA)

**Figure 1:**
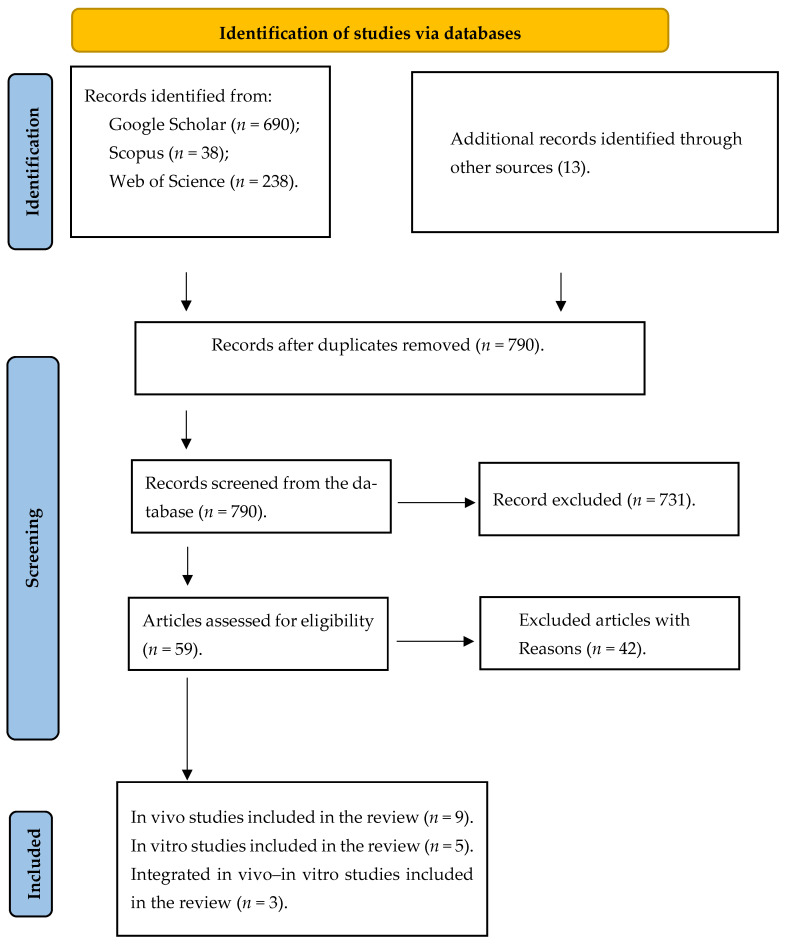
PRISMA 2020 flow diagram illustrating the study selection process for DDGS interventions in ruminant diets (2010–2025) [27].

This systematic review was conducted in accordance with the Preferred Reporting Items for Systematic Reviews and Meta-Analyses (Supplementary File S1) guidelines to ensure methodological transparency and reproducibility (Figure 1) [27]. The review protocol, including the study objectives, eligibility criteria, and planned approach for data synthesis, was developed prior to the initiation of the review process. The review protocol was developed a priori and fully defined before study selection and data extraction. Although the protocol was not registered in PROSPERO due to its limited scope for animal nutrition and CH_4_ mitigation interventions at the time of study initiation, all eligibility criteria, search strategy, and analytical procedures were strictly adhered to throughout the review process. No post hoc modifications were introduced. The primary objective of this review was to systematically identify, synthesize, and critically evaluate available evidence on the use of DDGS as a dietary strategy for mitigating enteric CH_4_ emissions in ruminant production systems.

### 2.2. Eligibility Criteria

Studies were considered eligible for inclusion if they involved ruminant species (cattle, sheep, or goats) and evaluated the use of DDGS as a dietary intervention, with reported outcomes on enteric CH_4_ emissions, GHG metrics, or CH_4_-related in vitro gas production. The review included in vivo feeding trials, in vitro rumen fermentation studies, and integrated in vivo–in vitro investigations. Only peer-reviewed original research articles published between 2010 and 2025 were considered to capture contemporary DDGS feeding systems and CH_4_ measurement methodologies. Studies were excluded if they involved non-ruminant species, did not include DDGS as a dietary treatment, lacked CH_4_-related outcomes, or were non-original publications such as reviews, meta-analyses, conference proceedings, or theses. Publications with inaccessible full texts or insufficient methodological detail were also excluded. The detailed inclusion and exclusion criteria are summarised in Table 1.

### 2.3. Information Sources and Search Strategy

A systematic literature search was conducted to identify relevant studies published between 2010 and 2025 using three major scientific databases: Google Scholar, Scopus, and Web of Science (Table 2). These databases were selected to ensure broad coverage of peer-reviewed literature in ruminant nutrition, CH_4_ mitigation, and feed evaluation. Searches were performed using database-specific search fields. In Google Scholar, searches were conducted across titles, abstracts, keywords, and the full text of indexed records, whereas Scopus searches were restricted to the TITLE-ABS-KEY fields, and Web of Science searches were conducted using the Topic (TS) field, which searches titles, abstracts, author keywords, and Keywords Plus. Search terms were structured around four key concepts: DDGS-related terms, methane/greenhouse gas terminology, ruminant livestock descriptors, and nutrient utilization parameters. Keywords within each concept were combined using the Boolean operator “OR”, while the different concepts were linked using “AND” to refine the search. Truncation and alternative terms (e.g., “DDGS”, “distillers’ grains”, “methane”, “CH_4_”, “digestibility”, “rumen fermentation”) were used to improve search sensitivity. The search strategy was iteratively refined to optimize sensitivity and specificity for relevant studies. In addition to the database search, manual citation tracking of the reference lists of relevant review and original research articles was conducted to identify additional eligible studies that were not captured through the electronic database searches. No language restrictions were applied during the initial database searches; however, only studies with accessible full texts and those that could be adequately translated were included in the review. The grey literature, including conference proceedings, theses, dissertations, book chapters, editorials, and other non-peer-reviewed publications, was not considered because the review aimed to synthesize evidence from peer-reviewed original research articles. Only peer-reviewed studies were considered to ensure methodological quality and consistency across the included evidence base.

### 2.4. Data Extraction and Management

Data were extracted using a standardized form. A predefined data extraction form was developed and applied to capture key study characteristics, including author(s), year of publication, country, experimental system (in vitro, in vivo, or integrated in vivo and in vitro approaches), animal species or rumen inoculum source, dietary composition, DDGS inclusion levels, and type or source of DDGS. In addition, outcome variables related to enteric CH_4_ emissions (e.g., CH_4_ production, yield, and intensity), DMI, nutrient digestibility, nitrogen utilisation, rumen fermentation parameters (e.g., VFAs and NH_3_-N), and animal performance indicators were systematically extracted where available. Titles, abstracts, full-text screening, data extraction, and risk-of-bias assessment were conducted independently by two reviewers, with discrepancies resolved through discussion and consensus. Where consensus could not be reached, a third independent reviewer was consulted to resolve disagreements and reach a final decision, thereby improving consistency and minimizing selection bias. The inter-reviewer agreement during title and abstract screening was high (Cohen’s κ = 0.82), indicating strong consistency in study selection. Prior to considering quantitative synthesis, the included studies were evaluated for methodological and clinical heterogeneity. Considerable heterogeneity was identified across several domains, including animal species (dairy cows, beef cattle, and sheep), experimental systems (in vivo and in vitro), DDGS source (corn, wheat, reduced-fat DDGS, and varying oil concentrations), inclusion levels (supplementation to approximately 40% of dietary DM), basal diet composition (high-forage, high-concentrate, and mixed diets), methane measurement techniques (GreenFeed systems, respiration chambers, SF_6_ tracer technique, and in vitro gas production systems), and outcome reporting units (CH_4_ production, CH_4_ yield, CH_4_ intensity, CH_4_ as a proportion of gross energy intake, and methane per unit of DMI or animal product). Due to substantial heterogeneity among the included studies in terms of experimental design, DDGS inclusion levels, dietary composition, animal species, and CH_4_ measurement techniques, a quantitative meta-analysis was not feasible. Because these sources of heterogeneity would violate the assumptions required for meaningful pooling of effect sizes and substantially reduce the interpretability of summary estimates, statistical synthesis was considered inappropriate. Instead, a qualitative synthesis approach was adopted. Extracted data were analyzed descriptively to identify patterns, consistencies, and variations in CH_4_ emissions and nutrient utilization responses associated with DDGS inclusion. Particular emphasis was placed on comparing outcomes between in vivo and in vitro systems, as well as evaluating the influence of DDGS inclusion level, chemical composition (especially ether extract content), and basal diet characteristics on CH_4_ mitigation responses. Where possible, results were interpreted in terms of CH_4_ production, CH_4_ yield (per unit intake), and CH_4_ intensity (per unit output) to provide a more comprehensive evaluation of CH_4_ responses.

### 2.5. Risk of Bias Assessment

The methodological quality and risk of bias of the included studies were assessed using the Systematic Review Centre for Laboratory Animal Experimentation (SYRCLE) risk-of-bias tool [28], which is specifically designed for animal-based intervention studies. Risk-of-bias assessment was independently conducted by two reviewers using the SYRCLE tool, with disagreements resolved through discussion and consensus. This framework was considered appropriate because the included studies comprised in vivo animal feeding trials, in vitro rumen fermentation experiments, and integrated in vivo–in vitro studies evaluating the effects of DDGS on CH_4_ emissions and nutrient utilization. The SYRCLE tool evaluates multiple domains of bias, including selection bias (sequence generation, baseline characteristics, and allocation concealment), performance bias (random housing and blinding of caregivers), detection bias (random outcome assessment and blinding of outcome assessors), attrition bias (incomplete outcome data), reporting bias (selective outcome reporting), and other sources of bias. Each study was subsequently classified as having low, high, or unclear risk of bias based on the level of methodological rigor and reporting transparency. To enhance objectivity and consistency, the risk-of-bias assessment was conducted independently by two reviewers, and any disagreements were resolved through discussion and consensus.

### 2.6. Data Synthesis

The included studies were synthesized qualitatively according to experimental system (in vivo, in vitro, and integrated studies), DDGS inclusion level, and the reported outcomes related to enteric CH_4_ emissions, nutrient utilization, rumen fermentation, and animal performance. A formal assessment of reporting bias due to missing results (e.g., publication bias) was not undertaken because the qualitative nature of the evidence synthesis and the heterogeneity of the included studies precluded meaningful assessment. Similarly, the certainty of the body of evidence was not formally assessed using the GRADE approach or any other evidence certainty framework.

## 3. Results

### 3.1. PRISMA Synthesis

A total of 966 records were identified through database searching, including Google Scholar (*n* = 690), Web of Science (*n* = 238), and Scopus (*n* = 38), with an additional 13 records identified through manual citation tracking. After removal of duplicates, 790 records remained and were screened based on title and abstract, of which 731 were excluded for not meeting the eligibility criteria. The full texts of 59 articles were assessed for eligibility, resulting in the exclusion of 42 studies primarily because DDGS was not evaluated as a dietary intervention, CH_4_-related outcomes were not reported, or the studies did not involve ruminants. A total of 17 studies met the inclusion criteria and were included in the final qualitative synthesis, comprising 9 in vivo and 8 in vitro studies. The included studies were published between 2012 and 2025, with publication frequency increasing markedly after 2019, indicating growing research interest in the use of DDGS for enteric CH_4_ mitigation. The study selection process is summarized in Figure 1.

### 3.2. Risk of Bias Assessment

Table 3 illustrates the risk of bias across the included studies; most domains related to baseline characteristics and incomplete outcome data were consistently assessed as low risk, indicating that experimental groups were generally comparable and that outcome reporting was mostly complete. In addition, several studies reported appropriate experimental designs (e.g., completely randomized and Latin square designs), supporting a low risk of bias in sequence generation. However, several domains were frequently classified as unclear risk, particularly allocation concealment, blinding of caregivers, and blinding of outcome assessors, due to limited reporting of these methodological details. Likewise, random housing and random outcome assessment were not consistently described across studies. These domains were frequently underreported across the included studies. Selective reporting bias was generally assessed as unclear, as most studies did not provide sufficient detail to confirm whether all measured outcomes were reported. Nevertheless, no strong evidence of systematic omission of key results was identified. Overall, the included studies demonstrated moderate methodological quality, with limitations primarily related to reporting completeness. Nevertheless, studies were not quantitatively weighted according to methodological quality, which may limit interpretation of the relative strength of evidence across included studies. Table 3 illustrates the risk of bias in the seventeen included studies.

### 3.3. Synthesized Findings

Methane responses in the in vitro studies are summarized in Table 4. Among the eight studies, CH_4_ production was reduced in five studies [30,35,36,37,38], whereas three studies reported no significant effect [8,33,34]. Responses in nutrient utilization were variable, with several studies reporting improved digestibility and altered rumen fermentation characteristics following DDGS supplementation [30,35,36,37,38].

The findings from the in vivo studies are presented in Table 5. Among the nine studies, six reported reductions in at least one CH_4_ metric, including CH_4_ production, CH_4_ yield, CH_4_ intensity, or CH_4_ expressed relative to gross energy intake, at DDGS inclusion levels ranging from 10% to 40% of diet DM [5,6,7,9,25,32]. In contrast, three studies reported no significant effect on absolute CH_4_ production [24,29,31]. Reductions in CH_4_ yield or intensity were reported more consistently than reductions in absolute CH_4_ production. Dry matter intake generally increased at low-to-moderate DDGS inclusion levels [5,6,9,25,31], remained unchanged in three studies [24,29,31], and decreased only at the highest inclusion level (40% DM) evaluated by Hünerberg et al. [32]. Improvements in crude protein digestibility were reported in five studies [5,6,7,24,32], whereas nitrogen utilization responses were inconsistent, with positive nitrogen balance reported in several studies [5,7,24] but increased urinary nitrogen excretion was observed at higher DDGS inclusion levels [32].

The combined in vivo and in vitro studies are summarized in Table 6. Across these studies, DDGS supplementation did not consistently reduce CH_4_ production but generally maintained methane emissions while producing modest changes in rumen fermentation and nutrient utilization [8,33,34]. Digestibility responses were dependent on inclusion level, with moderate DDGS supplementation generally improving nutrient digestibility, whereas higher inclusion levels reduced digestibility in some cases. Animal performance was largely unaffected, while rumen fermentation characteristics, including volatile fatty acids, ammonia-N, pH, and gas production, exhibited variable responses across studies.

### 3.4. Distribution of Studies by Countries

The geographical distribution of studies included in this systematic review shows that research on the use of DDGS for CH_4_ mitigation in ruminant systems was concentrated in a few countries. Studies included in this review were conducted primarily in the United States (*n* = 7), followed by Canada (*n* = 3) and Mexico (*n* = 2). Additional studies originated from China, Argentina, Austria, the United Kingdom, and Poland.

## 4. Discussion

### 4.1. Effects of DDGSs on Enteric Methane Emissions

The findings of this review indicate that DDGS influences enteric CH_4_ emissions indirectly through changes in dietary nutrient composition and rumen substrate availability rather than through direct inhibition of methanogenesis. Across the included studies, responses in absolute CH_4_ production were inconsistent, whereas reductions in CH_4_ yield and emission intensity were more frequently reported [5,7,24]. This suggests that potential benefits of DDGSs may lie in improving production efficiency rather than consistently reducing total CH_4_ emissions; however, this response was not observed uniformly across the included studies. The effects of DDGS on CH_4_ emissions are mainly associated with its nutrient profile, particularly its lipid concentration and the replacement of fermentable carbohydrates within the diet. Dietary lipids can reduce methanogenesis by decreasing the availability of fermentable organic matter and suppressing H_2_-producing microbial activity, thereby limiting the H_2_ available for CH_4_ synthesis [18]. In addition, replacing starch- or fiber-rich feed ingredients with DDGS alters ruminal fermentation pathways and substrate utilization patterns, which may further influence CH_4_ production [39]. From a bioenergetic perspective, the higher digestible energy density and metabolizable protein content of DDGS can improve feed utilization efficiency and support greater milk or meat production without a proportional increase in enteric CH_4_ production. Consequently, absolute CH_4_ emissions (e.g., L day^−1^) may remain unchanged or increase slightly as feed intake and production increase, whereas CH_4_ yield (e.g., g kg^−1^ DMI) and CH_4_ emission intensity (e.g., g kg^−1^ energy-corrected milk or live weight gain) decline because animal productivity increases at a faster rate than ruminal CH_4_ production. Despite these mechanisms, the evidence does not support DDGS as a consistent CH_4_ mitigation strategy. While several studies reported reductions in CH_4_ yield or CH_4_ emission intensity, others observed little or no effect on absolute CH_4_ emissions [8,24,29,31,33,34]. Such variability likely reflects differences in DDGS composition, dietary formulation, animal type, and production system. Therefore, any environmental advantage associated with DDGS may arise primarily from improved feed conversion efficiency and greater animal output per unit of CH_4_ emitted rather than from consistent reductions in total enteric CH_4_ production.

### 4.2. Effects of DDGSs on Feed Intake and Nutrient Utilization

The evidence synthesized in this review suggests that DDGSs may enhance feed intake and nutrient utilization under appropriate dietary conditions; however, these responses were not consistent across all studies. These responses are mainly attributable to its nutritional composition, particularly its high concentrations of digestible energy, crude protein, and rumen-undegradable protein, which increase the supply of metabolizable nutrients available for productive functions. Across the included studies, DDGS supplementation maintained or increased DMI while improving crude protein utilization, nitrogen retention, and feed efficiency [5,6,7,24,25]. Therefore, these findings suggest that DDGSs may replace conventional feed ingredients without compromising nutrient intake under some dietary conditions, although responses varied among studies. A consistent pattern emerging from the literature is that improvements in nutrient utilization were more pronounced than changes in feed intake. While DMI often remained relatively stable, enhanced nitrogen use efficiency and improved utilization of dietary energy were frequently reported. This response may be attributed to the high metabolizable protein content of DDGS, which enhances the utilization of absorbed nutrients for growth, milk synthesis, and other productive functions. Therefore, DDGSs may contribute additional nutrients to the diet and, under suitable dietary conditions, support more efficient nutrient utilization. However, the positive effects of DDGSs on nutrient utilization are influenced by inclusion level and dietary composition. Several studies reported reductions in fiber digestibility at higher inclusion levels [8,32], which may be linked to elevated dietary lipid concentrations and altered fiber characteristics. These changes can limit the degradation of structural carbohydrates, thereby reducing the efficiency of nutrient extraction from fibrous feeds. These findings suggest that the nutritional value of DDGS is more likely to be realized when dietary formulations balance its contribution to energy and protein supply with the maintenance of adequate fiber digestion and rumen function.

### 4.3. Effects of DDGSs on Rumen Fermentation Characteristics

Rumen fermentation responses to DDGS supplementation are determined by the manner in which DDGS alters nutrient supply to the ruminal microbial community. As a co-product rich in protein, digestible fiber, and residual lipids, DDGS modifies the balance of substrates available for microbial fermentation, thereby influencing fermentation end-products and nitrogen metabolism. Subsequently, changes in rumen fermentation are expected to reflect shifts in nutrient availability rather than a direct effect of DDGS on microbial activity. Across the studies included in this review, ruminal pH generally remained within physiological ranges following DDGS supplementation, suggesting that DDGSs can be incorporated into ruminant diets without compromising rumen stability under the dietary conditions evaluated [25,33]. Responses in VFA production were less consistent. Several studies reported reductions in the acetate-to-propionate ratio and alterations in microbial populations following DDGS inclusion [5,9,24], whereas others observed little change in total VFA concentration or individual VFA proportions [25]. Thus, such variation suggests that fermentation outcomes are influenced by the extent to which DDGS replaces starch-rich or fiber-rich ingredients within the diet.

Nitrogen metabolism also exhibited variable responses among studies. Given its relatively high crude protein content, DDGS can increase nitrogen supply within the rumen; however, changes in ammonia-N concentrations were inconsistent [25,33]. These differences likely reflect variation in protein degradability, microbial nitrogen demand, and the synchrony between nitrogen release and fermentable energy supply. At moderate inclusion levels, the additional nitrogen supplied by DDGSs may be incorporated more efficiently into microbial protein synthesis and productive functions, although this response varied among the included studies. However, when DDGS inclusion exceeds approximately 30% of dietary DM, crude protein intake may surpass microbial nitrogen requirements, resulting in increased ruminal ammonia absorption, hepatic urea synthesis, and greater urinary nitrogen excretion, as reported by Hünerberg et al. [32]. This shift from productive nitrogen utilization towards urinary nitrogen losses has important environmental implications because urinary urea is rapidly hydrolyzed to ammonia after excretion and may subsequently contribute to nitrous oxide emissions during manure storage and land application.

Similar inconsistencies were observed in vitro, where DDGS supplementation produced variable effects on VFA production and nitrogen utilization [30,35,36,38]. Differences between in vitro and in vivo responses should also be considered when interpreting the effects of DDGSs on rumen fermentation. Batch culture studies generally reported reductions in total gas production and, in some cases, total VFA production following DDGS supplementation [36,38], whereas in vivo studies frequently maintained or increased DMI while producing relatively minor changes in ruminal VFA concentrations [5,25]. These contrasting responses reflect the inherent differences between the two experimental systems. Static batch incubations are closed systems in which fermentation end-products accumulate because volatile fatty acids are neither absorbed across the rumen epithelium nor removed through digesta passage. The accumulation of fermentation products, together with declining pH during incubation, can progressively suppress microbial activity, resulting in lower gas production and fermentation rates. In contrast, the rumen is a dynamic environment where continuous feed intake, salivary buffering, absorption of VFAs across the rumen wall, and digesta passage maintain relatively stable fermentation conditions and support sustained microbial activity. Therefore, reductions in gas production or VFA concentration observed in batch culture systems should be interpreted cautiously, as they may overestimate the inhibitory effects of DDGSs on rumen fermentation compared with responses measured in vivo.

### 4.4. Effects of DDGSs on Animal Performance

Animal productivity is ultimately determined by the quantity of metabolizable nutrients available to support growth, lactation, and other productive functions. The nutritional characteristics of DDGS, particularly its concentrations of digestible energy and rumen undegradable protein, suggest that it may serve as a useful feed ingredient for improving nutrient supply in ruminant diets. Supplementation with DDGS was associated with positive or neutral production responses in several studies, although improvements in milk yield and growth performance were not consistently observed. More favorable responses were generally reported at inclusion levels between 0 and 20% of dietary DM [5,31]. These responses are consistent with established principles of ruminant nutrition, whereby increased metabolizable protein supply enhances amino acid availability and supports more efficient nutrient partitioning towards productive outputs [20,40]. Production responses were most pronounced when DDGS replaced feed ingredients of lower nutritional value. Under these circumstances, the additional energy and protein supplied by DDGS may improve the overall nutrient density of the diet, thereby supporting animal performance under appropriate dietary conditions [41,42]. However, increasing DDGS inclusion did not always produce further gains. At inclusion levels exceeding 30% of diet DM, productive responses became less predictable, with some studies reporting no additional benefit or even reduced performance [8,32]. This finding suggests that the relationship between DDGS inclusion and animal performance is not linear and that excessive inclusion may reduce the efficiency with which nutrients are utilized. From a production systems perspective, the potential value of DDGS lies in its capacity to improve the conversion of feed nutrients into animal products under suitable dietary conditions. Enhanced milk yield, growth rate, and feed efficiency increase productive output from a given quantity of feed resources, strengthening the economic and environmental sustainability of ruminant production. Accordingly, the available evidence suggests that DDGS is more likely to provide nutritional benefits when used to complement balanced diet formulation rather than when the inclusion level is simply maximized.

### 4.5. Influence of DDGS Inclusion Level, Source and Diet Composition

Variation in the responses observed throughout this review can be explained by the interaction among DDGS inclusion level, DDGS composition, and basal diet characteristics. Rather than exerting a uniform effect, DDGS influences ruminant production systems through modifications in nutrient supply, with the resulting outcomes dependent on how these changes interact with rumen function and animal nutrient requirements. Therefore, differences in CH_4_ emissions, nutrient utilization, rumen fermentation, and animal performance should be attributed to the combined effects of DDGS inclusion level, nutrient composition, and basal diet characteristics rather than to DDGS supplementation alone. The available studies suggest that responses become increasingly variable at higher DDGS inclusion levels; however, the evidence is insufficient to define a universal inclusion threshold. Across the available studies, inclusion levels between 0 and 20% of dietary DM were more frequently associated with favorable nutrient utilization, productive performance, and reductions in CH_4_ yield or emission intensity; however, these responses were not consistent across all studies [5,7,24]. Within this range, DDGS may enhance dietary energy and protein supply while maintaining rumen function, depending on its composition and the characteristics of the basal diet. In contrast, responses became less predictable when inclusion levels exceeded 30% of diet DM, with inconsistent effects reported for CH_4_ emissions, intake, digestibility, and productivity [8,32,36]. This shift suggests that nutritional benefits associated with DDGS can be offset when dietary modifications begin to constrain ruminal digestion processes.

Differences in DDGS sources further contribute to response variability. The chemical composition of DDGS is influenced by the grain used during ethanol production and by processing conditions, resulting in considerable variation in lipid, fiber, and protein concentrations. For example, corn-based DDGS typically contains higher residual lipid concentrations than wheat-based DDGS, which may alter hydrogen utilization patterns within the rumen and influence CH_4_ formation [42]. At the same time, elevated lipid concentrations may reduce cellulolytic activity, limiting fiber degradation when DDGS is incorporated at excessive levels [18]. These compositional differences highlight the importance of evaluating DDGS according to its nutritional characteristics rather than treating it as a single, uniform feed ingredient. The response to DDGS is also strongly influenced by the characteristics of the basal diet. In forage-based systems, DDGS often replaces a portion of the structural carbohydrates supplied by forage ingredients, increasing the likelihood of changes in fiber digestion and fermentation patterns [6,32]. In contrast, diets containing greater proportions of concentrate may accommodate DDGS more readily because the ingredient complements existing energy sources without substantially altering the fiber fraction. Consequently, similar DDGS inclusion levels can produce markedly different outcomes depending on the nutritional composition of the diet into which it is incorporated.

### 4.6. Limitations and Future Directions

The interpretation of DDGS effects on enteric CH_4_ emissions and nutrient utilization is constrained by several interrelated limitations within the current evidence base. A key limitation is that DDGS cannot be considered a uniform dietary component, as its chemical composition varies according to grain source and processing conditions. This variability in lipid, fiber, and protein fractions interacts with differences in inclusion level and basal diet composition, making it difficult to isolate the mechanisms responsible for changes in CH_4_ emissions and nutrient utilization. As previously stated, the absence of consistent responses across studies reflects this interaction, particularly where opposing effects of lipid and fiber fractions influence H_2_ availability and fermentation pathways. Further complexity arises from inconsistencies in how CH_4_ emissions are reported. Expressing CH_4_ as production, yield, or intensity captures different biological processes, and the lack of standardization limits direct comparisons across studies. Thus, this issue is closely linked to the observation that DDGS does not appear to act as a direct CH_4_ inhibitor but rather modifies rumen fermentation and nutrient utilization in ways that may alter CH_4_ outcomes indirectly. In addition, most available studies are short-term and conducted under controlled conditions, which may not fully represent longer-term microbial adaptation or production responses under practical feeding systems. Future research would benefit from approaches that explicitly integrate CH_4_ emissions with rumen fermentation dynamics and nutrient utilization within the same experimental framework. Therefore, establishing clear inclusion thresholds under varying dietary conditions would improve the interpretation of non-linear responses observed at higher inclusion levels. Longer-term in vivo studies conducted under production conditions would provide insight into adaptation effects and the stability of responses over time. Furthermore, expanding the evidence base across diverse production systems, particularly those reliant on low-quality forages, would further clarify the role of DDGS in improving feed efficiency while managing CH_4_ emissions.

## 5. Conclusions

This systematic review demonstrated that DDGS supplementation did not consistently reduce absolute CH_4_ production in ruminants, although reductions in CH_4_ yield and emission intensity were more frequently reported across the included studies. The responses to DDGS supplementation varied according to inclusion level, DDGS source, basal diet composition, and nutrient substitution patterns, highlighting the importance of considering these factors when formulating ruminant diets. Across the available evidence, inclusion levels of up to 20% of dietary DM were more frequently associated with favorable production responses and reductions in CH_4_ yield or emission intensity; however, these responses were not observed consistently across all studies. Overall, the current evidence suggests that DDGS should not be regarded as a dedicated methane mitigation additive. Rather, it may serve as a multifunctional feed ingredient that can improve production efficiency and, under appropriate dietary conditions, contribute to reductions in methane intensity. Nevertheless, the magnitude and direction of these responses remain dependent on dietary formulation and experimental conditions. Future research should prioritize long-term in vivo studies, evaluation of microbial adaptation, and standardized CH_4_ measurement and reporting protocols to improve comparability among studies and strengthen the evidence base.

## Figures and Tables

**Table 1:** Inclusion and exclusion criteria for this systematic review.

Criterion	Inclusion	Exclusion
Publication type	Peer-reviewed journal articles reporting original research	Reviews, meta-analyses, book chapters, conference proceedings, editorials, theses
Language	English and non-English publications (where translation was feasible)	Publications for which full text could not be accessed or adequately translated
Population	Ruminant livestock (cattle, sheep, goats)	Non-ruminant species
Intervention	Diets containing distillers dried grains with solubles (DDGSs)	Studies where DDGS is not included as a dietary treatment or its individual contribution could not be isolated
Outcomes	Enteric methane (CH_4_) or GHG metrics; methane-related in vitro gas production	No methane or GHG-related outcomes
Study design	In vivo animal feeding trials, in vitro rumen fermentation studies, and integrated in vivo–in vitro studies	Modelling or observational studies without intervention
Publication period	2010–2025	Before 2010

**Table 2:** Search strings used for the systematic literature search.

Database	Search String
Google Scholar	(“distillers dried grains with solubles” OR DDGS OR “dried distillers’ grains” OR “distillers’ grains”) AND (“enteric methane” OR methane OR CH_4_ OR “methane emission” OR “greenhouse gas” OR GHG) AND (ruminant OR cattle OR sheep OR goat) AND (“nutrient utilization” OR digestibility OR “feed intake” OR “dry matter intake” OR “rumen fermentation” OR “volatile fatty acids”)
Scopus	TITLE-ABS-KEY ((“distillers dried grains with solubles” OR DDGS OR “dried distillers’ grains” OR “distillers’ grains”) AND (ruminant OR cattle OR cow OR dairy OR beef OR sheep OR goat) AND (methane OR “enteric methane” OR CH4 OR “methane emissions”) AND (“nutrient utilization” OR digestibility OR “feed intake” OR “dry matter intake” OR “rumen fermentation” OR “volatile fatty acids”))
Web of Science	TS = ((“distillers dried grains with solubles” OR DDGS OR “dried distillers’ grains” OR “distillers’ grains”) AND (ruminant OR cattle OR cow OR dairy OR beef OR sheep OR goat) AND (methane OR “enteric methane” OR CH_4_ OR “methane emissions”) AND (“nutrient utilization” OR digestibility OR “feed intake” OR “dry matter intake” OR “rumen fermentation” OR “volatile fatty acids”))

**Table 3:** Risk of bias ratings for included studies.

Study	Sequence Generation	Baseline Characteristics	Allocation Concealment	Random Housing	Blinding of Caregivers	Random Outcome Assessment	Blinding of Outcome Assessor	Incomplete Outcome Data	Selective Reporting	Other Bias
Benchaar et al. (2013) [5]	Low	Low	Unclear	Low	Unclear	Low	Unclear	Low	Unclear	Low
Bernier et al. (2012) [29]	Unclear	Low	Unclear	Low	Unclear	Unclear	Unclear	Low	Low	Low
Castillo-Lopez et al. (2017) [24]	Low	Low	Unclear	Low	Unclear	Low	Unclear	Low	Low	Low
Cobos-Peralta et al. (2018) [30]	Low	Low	Unclear	Low	Low	Low	Unclear	Low	Low	Low
Curzaynz-Leyva et al. (2020) [8]	Low	Low	Unclear	Low	Unclear	Low	Unclear	Low	Low	Low
Fincham et al. (2025) [31]	Low	Low	Unclear	Low	Unclear	Low	Unclear	Low	Low	Low
Gere et al. (2022) [6]	Unclear	Low	Unclear	Low	Unclear	Low	Unclear	Low	Low	Low
Hünerberg et al. (2013) [32]	Low	Low	Unclear	Low	Unclear	Low	Unclear	Low	Low	Low
Keomanivong et al. (2017) [33]	Low	Low	Unclear	Low	Unclear	Low	Unclear	Low	Low	Low
Keomanivong et al. (2018) [34]	Low	Low	Unclear	Low	Unclear	Low	Unclear	Low	Low	Low
Khiaosa-ard et al. (2015) [35]	Low	Low	Unclear	Low	Low	Low	Unclear	Low	Low	Low
Knoell et al. (2024) [7]	Low	Low	Unclear	Low	Low	Low	Unclear	Low	Low	Low
Miśta et al. (2014) [36]	Low	Low	Unclear	Low	Low	Low	Unclear	Low	Low	Low
Shreck et al. (2021) [9]	Low	Low	Unclear	Low	Unclear	Low	Unclear	Low	Low	Low
Smith et al. (2020) [37]	Low	Low	Unclear	Low	Low	Low	Unclear	Low	Low	Low
Wang et al. (2025) [25]	Low	Low	Unclear	Low	Unclear	Low	Unclear	Low	Low	Low
Xue et al. (2025) [38]	Low	Low	Unclear	Low	Low	Low	Unclear	Low	Low	Low

**Table 4:** Summary of in vitro studies evaluating the effects of DDGSs on methane emissions and nutrient utilization in ruminants.

Authors	Forage Source	Study Design	Key Findings	Country
Cobos-Peralta et al. (2018) [30]	15:85 forage: concentrate diet (maize stover + concentrate)	0%, 20%, 40% DDGS (DM basis)	CH_4_ (cumulative): ↓ (20%); ↔ (40% vs. control); CH_4_ proportion: ↓; CO_2_: ↑ (40%); Total gas: ↑ (40%); Digestibility: ↓; VFA, pH: ↔	Mexico
Khiaosa-ard et al. (2015) [35]	Meadow hay + concentrate (48:52)	DDGS at ~19.5–25% DM; GSM fortified at 0, 1, 5, 10, 20% within DDGS	CH_4_: ↓ (≥5% GSM); CH_4_ (g/NDF): ↓; NH_3_: ↑ (DDGS); CP degradability: ↑; OM/DM degradability: ↓ (with GSM); Protozoa: ↓; Fungi: ↓	Austria
Miśta et al. (2014) [36]	Concentrate-based substrate (cereal + oilseed meals replaced with DDGSs)	DDGS at 0, ~10, 15, 20, and 100% replacement of concentrate ingredients	CH_4_: ↓; Total gas: ↓; VFA (total): ↓; VFA profile (acetate, propionate, butyrate): ↔; Ammonia: ↓; pH: ↔; Fermentation efficiency: ↔	Poland
Smith et al. (2020) [37]	Bermudagrass (Coastal & Tifton 85)	DDGS inclusion: 0%, 12.5%, and 50% (forage: DDGS = 100:0, 87.5:12.5, 50:50)	CH_4_: ↓ (linear); Digestibility: ↑; Fibre digestibility: ↑	USA
Xue et al. (2025) [38]	Grass silage: concentrate (70:30) basal substrate	DDGS at 10, 20, and 30% DM (replacing the concentrate fraction)	CH_4_ (mL): ↓; CH_4_/dDM: ↓ (mainly at 100 g/kg); Gas production: ↓; Digestibility (DM): ↓; NH_3_-N: ↑; VFA (total): ↔; A:P ratio: ↓	UK

Abbreviations: A:P ratio, acetate-to-propionate ratio; CH_4_, methane; CO_2_, carbon dioxide; CP, crude protein; DDGS, dried distillers’ grains with solubles; DM, dry matter; GSM, grape seed meal; NDF, neutral detergent fiber; NH_3_, ammonia; NH_3_-N, ammonia nitrogen; OM, organic matter; pH, potential of hydrogen; VFA, volatile fatty acids.

**Table 5:** Summary of in vivo studies evaluating DDGS supplementation in ruminants.

Authors	CH_4_ Measurement Method	Animal	Forage Source	Study Design	Key Findings	Country
Benchaar et al. (2013) [5]	Open-circuit respiration chamber technique	Lactating Holstein cows	Alfalfa silage, corn silage, timothy hay (TMR)	0, 10, 20, and 30% DM; DDGS-replaced corn and soybean meal were evaluated using a triplicated 4 × 4 Latin square design with 35-day experimental periods.	CH_4_: ↓; CH_4_/GEI: ↓; CH_4_/DMI: ↓; DMI: ↑; Digestibility (DM, OM, GE): ↓; CP digestibility: ↑; N utilization: ↓ (efficiency) but ↑ (productive); Milk yield: ↑; A:P ratio: ↓	Canada
Bernier et al. (2012) [29]	Sulphur hexafluoride (SF_6_) tracer gas technique	Mature beef cows (dry)	Low-quality grass hay + oat straw	0, 10, 20% DDGS (DM basis; protein supplementation), using a completely randomized design over a 140-day experimental period.	CH_4_ (L/d): ↔; CH_4_ (%GEI): ↓ (at 20%); DMI: ↔; CP intake: ↑; N status (SUN): ↑; CH_4_: ↓	Canada
Castillo-Lopez et al. (2017) [24]	Indirect calorimetry using headbox respiration chambers	Lactating Holstein cows	Corn silage, alfalfa hay, grass hay (TMR)	0%; 20% DDGS; 20% reduced-fat DDGS; 10% DDGS + 10% reduced-fat DDGS (DM basis; replaced corn & soybean meal) were evaluated using a 4 × 4 Latin square design over four 28-day experimental periods.	CH_4_ (L/d): ↔; CH_4_/DMI: ↓ (tendency); DMI: ↑; N utilization: ↑ (↓ rumen NH_3_); Microbial shifts: ↑ Firmicutes, ↓ Bacteroidetes	USA
Fincham et al. (2025) [31]	Headbox-type indirect respiration calorimeters (indirect calorimetry)	Lactating Jersey cows	TMR	DDGS at 0%, 6.5%, and 13% replacing starch, NDF, and forage NDF. Treatments were evaluated using a triplicated 4 × 4 Latin square design over four 28-day experimental periods.	H_4_: ↔; DMI: ↑ (reduced fiber diet); Milk yield: ↑ (RF diet); Digestibility: slight ↓ (RS diet); Manure CH_4_: ↔; Total manure CH_4_ potential: ↑ (RF, RS); Energy utilization: ↔	USA
Gere et al. (2022) [6]	Sulphur hexafluoride (SF_6_) tracer gas technique	Adult sheep	Rhodes grass hay (low-quality forage)	Hay alone vs. Hay + DDGS (64:36 DM; supplementation). Treatments were evaluated using a changeover design over two 27-day experimental periods.	H_4_ (g/d): ↓; CH_4_/DMI: ↓; Ym: ↓; DMI: ↑; Digestibility: ↑; CP: ↑; NDF: ↓	Argentina
Hünerberg et al. (2013) [32]	Open-circuit respiration chambers	Growing beef heifers	Barley silage-based high-forage diet	DDGS at 40% DM: CDDGS, WDDGS, WDDGS + oil (vs. control). Using a replicated 4 × 4 Latin square design over four 21-day experimental periods.	CH_4_ (g/d): ↓ (all DDGS); CH_4_/DMI: ↓ (CDDGS, WDDGS + oil); CH_4_/DMI: ↔ (WDDGS); DMI: ↓ (CDDGS, WDDGS); Digestibility (DM, OM): ↓ (CDDGS, WDDGS + oil); CP digestibility: ↑; N excretion: ↑	Canada
Knoell et al. (2024) [7]	Headbox-type indirect calorimetry	Lactating Jersey cows	Corn silage + alfalfa hay (replaced with straw + DDGS in TMR)	DDGS at 0, 6.0, 12.1, 18.1% DM (with straw; replaced alfalfa hay). The study used a triplicated 4 × 4 Latin square, consisting of four 35-day experimental periods (31-day adaptation and 4-day collection).	CH_4_ (L/d): ↓; CH_4_/DMI: ↓; CH_4_/ECM: ↓; DMI: ↔; Milk yield: ↔; CP digestibility: ↑; N balance: ↑; Digestibility (DM, OM): ↔; Rumination: ↓	USA
Shreck et al. (2021) [9]	GreenFeed CH_4_ measurement	Beef steers (British-cross)	Low-quality bluestem hay (≈4.6% CP)	Protein supplementation: DDGS (0.41% BW) vs. cottonseed meal (0.29% BW) vs. control (no supplement). Using a three-period crossover design with 28-day experimental periods.	CH_4_ (g/d): ↑ (CSM, DDGS vs. control); CH_4_ (%GEI): ↓ (lowest in DDGS); DMI: ↑ (with supplementation); Digestibility: ↑ (DDGS); CO_2_: ↑; VFA: acetate ↓, propionate ↑ (DDGS); A:P ratio: ↓; Energy efficiency: ↑ (lower Ym)	USA
Wang et al. (2025) [25]	Open-circuit respiratory calorimetry system	Dorper × Thin-tailed Han ewes	Corn stalk–based total mixed ration	DDGS at 15% diet replacement (protein source substitution). Treatments were evaluated using a CRD.	CH_4_ (L/d): ↓; CH_4_/DMI: ↓; DMI: ↑; Digestibility (DM): ↑; CP digestibility: ↔; Energy (GE, DE, ME, NE): ↑; Energy efficiency: ↑; VFA: ↔; A:P ratio: ↔; Methanobrevibacter: ↓	China

Abbreviations: A:P ratio, acetate-to-propionate ratio; CDDGS, corn dried distillers’ grains with solubles; CH_4_, methane; CO_2_, carbon dioxide; CP, crude protein; CRD, completely randomized design; CSM, cottonseed meal; DDGS, dried distillers’ grains with solubles; DE, digestible energy; DM, dry matter; DMI, dry matter intake; ECM, energy-corrected milk; GE, gross energy; GEI, gross energy intake; ME, metabolizable energy; N, nitrogen; NDF, neutral detergent fiber; NE, net energy; NH_3_, ammonia; RF diet, reduced-forage diet; RS diet, reduced-starch diet; SF_6_, sulfur hexafluoride; SUN, serum urea nitrogen; TMR, total mixed ration; VFA, volatile fatty acids; WDDGS, wheat dried distillers’ grains with solubles; Ym, methane conversion factor.

**Table 6:** Summary of in vivo and in vitro studies evaluating DDGS supplementation in ruminants.

Authors	Animal	Forage Source	Study Design	Key Findings	Country
Curzaynz-Leyva et al. (2020) [8]	Growing lambs (native Mexican)	Corn stover-based diet (with concentrate)	0, 20, 40% DDGS (DM basis; replaced grain, protein, and part of forage)	CH_4_ (in vitro): ↔; CO_2_: ↑ (40%); Total gas: ↑ (40%); DMI: ↔; ADG: ↔; Digestibility: ↑ (20%), ↓ (40%); VFA: ↓ (20%); pH: ↑ (20%); Carcass: ↔	Mexico
Keomanivong et al. (2017) [33]	Cannulated Holstein steers	Grass–legume hay + corn silage (high-concentrate diet)	DDGS at 20% vs. 40% DM; combined with coarse- vs. fine-rolled corn	CH_4_ (in vitro): ↔; Gas production: ↔; DMI: ↔; pH: ↔ (time < 5.5 ↑ at 40%); NH_3_: ↑ (20%); VFA: minor shifts; Enzyme activity: ↑/↓ (dose-dependent)	USA
Keomanivong et al. (2018) [34]	Cannulated Holstein steers	Corn silage (20% DM; high-concentrate diet)	25% DDGS (low-oil: 4.5% vs. moderate-oil: 7.9%; corn vs. barley-based diets)	CH_4_ (in vitro): ↔; CO_2_: ↔; DMI: ↔; pH: ↔; VFA: ↔; NH_3_: ↔; Enzyme activity: ↑/↓ (treatment-dependent)	USA

Abbreviations: ADG, average daily gain; CH_4_, methane; CO_2_, carbon dioxide; DDGS, dried distillers’ grains with solubles; DM, dry matter; DMI, dry matter intake; NH_3_, ammonia; pH, potential of hydrogen; VFA, volatile fatty acids.

## Data Availability

The data included in this systematic review were retrieved from Scopus, Web of Science, and Google Scholar based on the search strings provided.

## Supplementary Materials

The following supporting information can be downloaded at: https://www.mdpi.com/article/10.3390/ani16152427/s1, File S1. PRISMA 2020 Checklist [43].

## Abbreviations

The following abbreviations are used in this manuscript:

ADG	Average Daily Gain
A:P ratio	Acetate-to-Propionate Ratio
BW	Body Weight
CDDGSs	Corn Dried Distillers’ Grains with Solubles
CH_4_	Methane
CO_2_	Carbon Dioxide
CP	Crude Protein
CRD	Completely Randomized Design
CSM	Cottonseed Meal
DDGSs	Dried Distillers’ Grains with Solubles
DE	Digestible Energy
DM	Dry Matter
DMI	Dry Matter Intake
ECM	Energy-Corrected Milk
GE	Gross Energy
GEI	Gross Energy Intake
GHG	Greenhouse Gas
GSM	Grape Seed Meal
H_2_	Hydrogen
ME	Metabolizable Energy
N	Nitrogen
NDF	Neutral Detergent Fiber
NE	Net Energy
NH_3_	Ammonia
OM	Organic Matter
PRISMA	Preferred Reporting Items for Systematic Reviews and Meta-Analyses
RF diet	Reduced-Forage Diet
RS diet	Reduced-Starch Diet
Rusitec	Rumen Simulation Technique
SF_6_	Sulfur Hexafluoride
SUN	Serum Urea Nitrogen
TMR	Total Mixed Ration
VFA	Volatile Fatty Acids
WDDGS	Wheat Dried Distillers’ Grains with Solubles
Ym	Methane Conversion Factor
